# Identification of functionally connected multi-omic biomarkers for Alzheimer’s disease using modularity-constrained Lasso

**DOI:** 10.1371/journal.pone.0234748

**Published:** 2020-06-17

**Authors:** Linhui Xie, Pradeep Varathan, Kwangsik Nho, Andrew J. Saykin, Paul Salama, Jingwen Yan

**Affiliations:** 1 Department of Electrical and Computer Engineering, Indiana University Purdue University Indianapolis, Indianapolis, Indiana, United States of America; 2 Department of Radiology and Imaging Sciences, School of Medicine, Indiana University School of Medicine, Indianapolis, Indiana, United States of America; 3 Department of BioHealth Informatics, Indiana University Purdue University Indianapolis, Indianapolis, Indiana, United States of America; Niigata University, JAPAN

## Abstract

Large-scale genome wide association studies (GWASs) have led to discovery of many genetic risk factors in Alzheimer’s disease (AD), such as *APOE*, *TOMM40* and *CLU*. Despite the significant progress, it remains a major challenge to functionally validate these genetic findings and translate them into targetable mechanisms. Integration of multiple types of molecular data is increasingly used to address this problem. In this paper, we proposed a modularity-constrained Lasso model to jointly analyze the genotype, gene expression and protein expression data for discovery of functionally connected multi-omic biomarkers in AD. With a prior network capturing the functional relationship between SNPs, genes and proteins, the newly introduced penalty term maximizes the global modularity of the subnetwork involving selected markers and encourages the selection of multi-omic markers with dense functional connectivity, instead of individual markers. We applied this new model to the real data collected in the ROS/MAP cohort where the cognitive performance was used as disease quantitative trait. A functionally connected subnetwork involving 276 multi-omic biomarkers, including SNPs, genes and proteins, were identified to bear predictive power. Within this subnetwork, multiple trans-omic paths from SNPs to genes and then proteins were observed. This suggests that cognitive performance deterioration in AD patients can be potentially a result of genetic variations due to their cascade effect on the downstream transcriptome and proteome level.

## Introduction

Alzheimer’s disease (AD) is the most common form of brain dementia characterized by the gradual loss of memory and other cognitive function. With rapidly increasing aging population, AD is drawing more and more attention in the United States and around the world [1]. Unfortunately, the underlying mechanism of AD remains largely unknown and no clinically validated drug is available for disease treatment and prevention. Recent large-scale genome wide association studies (GWASs) have led to discovery of many genetic risk factors associated with AD, such as *APOE*, *TOMM40* and *CLU*. However, they are mostly individual markers, possibly without functional interactions, which presents difficulties to validate these findings and to relate them to downstream biology [2, 3]. Therefore, it remains a challenge to translate them into targetable mechanisms related to disease pathogenesis. Novel biomarkers are in need that can potentially serve as targets in the future therapeutic interventions.

Recently, there is a substantial increase of multi-omic data in AD. Example projects include the Alzheimer’s Disease Neuroimaging Initiative (ADNI) [4] and the Religious Orders Study and Memory and Aging Project (ROSMAP) [5]. Instead of limiting their perspective to a single data type, these data collections create a molecular landscape spanning the genome, transcriptome, proteome and metabolome. Coupling with various biological networks (e.g., protein-protein interaction (PPI) network), these data provides a valuable resource with rich content and opens numerous opportunities for more comprehensive analyses of AD. These multi-omic data has been increasingly recognized to be a potential key enabler of novel biomarker discovery [6, 7]. It not only allows us to examine the disease from different molecular scales, but also provides insights into their interactions which is critical for translation of genetic findings into targetable mechanism.

In this paper, we proposed a modularity-constrained Lasso model to jointly analyze the genotype, gene expression and protein expression data for discovery of functionally connected multi-omic biomarkers in AD. We constructed a network between SNPs, genes and proteins to capture their functional relationship and used it as a priori in the proposed model. Based on this, the newly introduced penalty term maximizes the global modularity of the subnetwork involving selected markers and encourages the selection of multi-omic markers with not only predictive power but also dense functional connectivity evidenced in the prior knowledge. We applied this new model to the real data collected in the ROS/MAP cohort and used the cognitive performance as disease quantitative trait. As a result, we identified a functionally connected subnetwork involving 276 multi-omic biomarkers, including SNPs, genes and proteins. Within this subnetwork, we observed a plenty number of paths cutting across multiple molecular types, from SNPs to genes and then proteins. This suggests that cognitive performance deterioration in AD patients can be potentially a result of genetic variations due to their cascade effect on the downstream transcriptome and proteome level. Such connected pattern can help improve not only the reliability of identified biomarkers, but also their replicability and interpretability.

## Data and method

### Study sample

All the data analyzed were obtained from the Religious Orders Study (ROS) and Memory and Aging Project (MAP). It was launched by Rush University to build a cohort from religious communities to measure the progression of amnestic mild cognitive impairment (MCI, a prodromal stage of AD) and early probable AD. The combined ROS/MAP cohort includes around 600 participants under age 90, which constitute a very rich repository of multi-modal data including GWAS data, whole genome sequencing (WGS) data, cognitive, behavioral and clinical data. The more detailed description could be found in [5]. In this paper, GWAS genotype data and quality controlled RNA-Seq gene expression and protein expression data collected from prefrontal cortex tissue in the brain were downloaded. To perform the proposed joint analysis, only subjects with all three types of data were included. In total, we have 262 subjects with full set of data, including 115 cognitive normals (CN), 67 MCIs and 80 AD patients. The detailed demographic information can be found in Table 1.

**Table 1:** Demographic information of the ROS/MAP participants included in this study.

Dignosis	CN	MCI	AD
Subject Number	115	67	80
ROS/MAP	69/46	27/40	40/40
Male/Female	51/64	28/39	31/49
Education(mean± std.)	16.9 ± 3.5	16.6 ± 3.3	16.9 ± 3.8
Age(mean± std.)	83.0 ± 4.7	85.0 ± 4.2	86.3 ± 3.7

### GWAS genotype data preparation

ROS/MAP samples were genotyped on the Affymetrix GeneChip 6.0 platform [8]. We performed sample and SNP quality control procedures on GWAS data (SNP call rate<95%, Hardy-Weinberg equilibrium test *p* < 10^−6^ in controls, and frequency filtering MAF<1%) were performed. After the standard quality control procedures for genetic markers and subjects, only non-Hispanic Caucasian participants were selected by clustering with CEU (Utah residents with Northern and Western European ancestry from the CEPH collection) + TSI (Toscani in Italia) populations using HapMap 3 genotype data and the multidimensional scaling (MDS) analysis [9]. Un-genotyped SNPs were imputed using MaCH and the 1000 Genomes Project as a reference panel [10]. Final SNP data used for the analysis is coded as the number of minor alleles.

### RNA-Seq gene expression preparation

RNA-Seq gene expression data in the ROS/MAP cohort were collected from the prefrontal cortex tissue in the brain. The RNA-Seq data were recently reprocessed in parallel with other AMP-AD RNAseq datasets, and this second version of the data were downloaded for our subsequent analysis. The input data for the RNA-Seq reprocessing effort was aligned reads in bam files that were converted to fastq using the Picard SamToFastq function. Fastq files were re-aligned to the reference genome using STAR with twopassMode set as Basic. Gene counts were computed for each sample by STAR by setting quantMode as GeneCounts. These gene level counts further went through normalization and adjustment to remove the effects of relevant factors such as age, gender, education, batch, RNA integrity number (RIN) and postmortem interval (PMI). Detailed reprocessing and normalization steps can be found in the AMP-AD knowledge portal (https://www.synapse.org/#!Synapse:syn9702085/).

### Protein expression data preparation

Selected reaction monitoring (SRM) proteomics was performed using frozen tissue from dorsolateral prefrontal cortex (DLPFC). The samples were prepared for LC-SRM analysis using standard protocol as described in [11, 12]. All the data were manually inspected to ensure correct peak assignment and peak boundaries. The abundance of endogenous peptides was quantified as a ratio to spiked-in synthetic peptides containing stable heavy isotopes. The “light/heavy” ratios were log2 transformed and shifted such that median log2-ratio is zero. Normalization adjusted for differences in protein amounts between the samples. During that normalization, the log2-ratios were shifted for each sample to make sure the median is set at zero. Detailed processing steps can be found in the AMP-AD knowledge portal (https://www.synapse.org/#!Synapse:syn8456629). Using the regression weights derived from the cognitive normal participants, peptide abundance data were further adjusted to remove the effects of age, gender, education, PMI and batch.

### Selection of SNPs, genes and proteins

**Fig 1:**
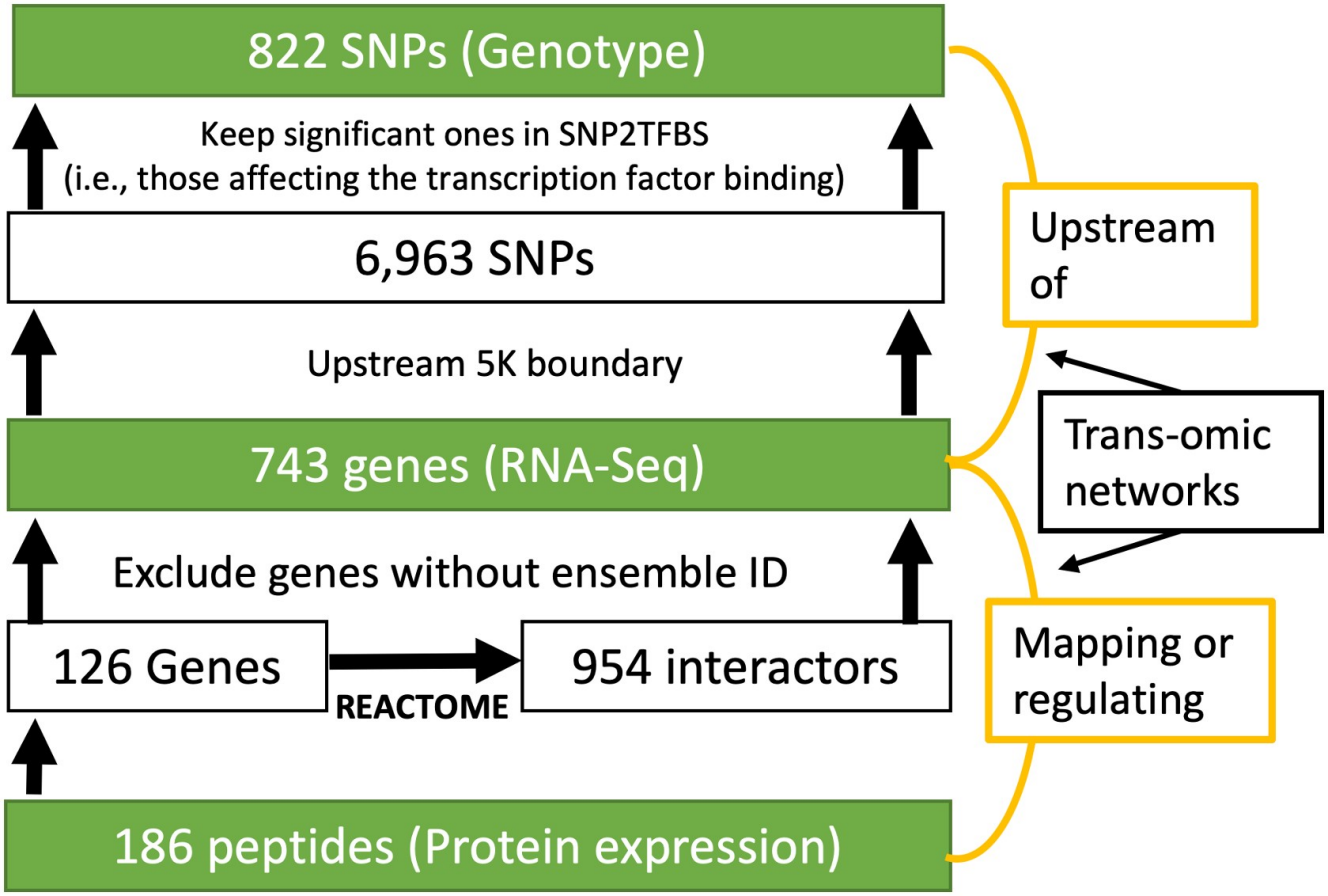
The selection of SNPs in upstream 5K boundary for each gene.

We focused our analysis on a set of SNPs, genes and proteins with known functional connections. Though we have genome-wide genotype and transcriptome-wide gene expression data available in the ROS/MAP cohort, only a limited number of proteins are measured, which forms a bottleneck for the joint data analysis. To address this problem, we took a bottom-up approach where proteins measured in the prefrontal cortex were used as seeds to select a subset of relevant SNPs and genes for subsequent analysis. As shown in Fig 1, in the proteomic level, abundance level of 186 peptides, corresponding to 126 unique genes, were measured in the ROS/MAP cohort. In the functional interaction network obtained from the REACTOME database, these 126 genes were found to interact with 954 genes and these interactions were all manually curated from known pathways [13]. Among these 1080 (=126+954) candidate genes, 743 of them without missing RNA-seq data were included to represent the transcriptomic level. Of note, we did not further filter these genes based on their differential expression in AD. In the genomic level, we identified SNPs located on the upstream of these 743 genes within the boundary of 5K. To ensure the functional connection of selected SNPs and the downstream genes, we included only SNPs significantly affecting the transcription factor binding activity, based on the SNP2TFBS database [14]. These relationships between SNPs, genes and proteins/peptides are used to build a trans-omic functional interaction network to guide the search of functionally connected biomarkers in the subsequent analysis.

### Memory outcomes

Cognitive performance of participants in the ROS/MAP cohort was estimated through the mini mental state examination, a standardized screening measure for collecting 30 items in related with dementia [15, 16]. This score ranges from 0 to 30, and is scaled to quantify the severity of dementia. In this study, we used this memory test score as the AD quantitative trait for biomarker discovery. Using the regression weights derived from the cognitive normal participants, the memory score is adjusted to remove the effect of age, sex and education.

### Modularity-constrained Lasso

Throughout this section, we write matrices as boldface uppercase letters and vectors as boldface lowercase letters. Given a matrix **M** = (*m*_*ij*_), its *i*-th row and *j*-th column are denoted as **m**^*i*^ and **m**_*j*_ respectively. Let **X** = [***x***_1_, ***x***_2_, …, ***x***_*n*_]^*T*^ be the multi-omic features as predictors and **y** = [*y*_1_, *y*_2_, …, *y*_*n*_]^*T*^ be the disease quantitative trait as outcome (i.e., cognitive performance). Here, $x_{j}\subseteq R^{p}$ is a concatenated vector of genotype, gene expression and protein expression data for *j*-th subject.

The least absolute shrinkage and selection operator (Lasso) is a shrinkage and selection method for linear regression [17]. It minimizes the usual sum of squared errors with a bound on the sum of the absolute values of the coefficients, which is also known as L1 norm (Eq 1).

$$\begin{array}{c} \min_{w}{∥y-Xw∥}^{2}{\;\text{s}.\text{t}.\;∥w∥}_{1}=\sum_{j=1}^{p}|w_{j}|\leq t \end{array}$$ (1)

With this constraint, Lasso aims to minimize the number of selected features, which significantly improved the interpretability of results compared to traditional linear regression, where almost all features are considered to be outcome-relevant with non-zero weight. However, when dealing with a group of highly correlated features, L1 norm penalty will result in a random selection. In this case, multiple runs of Lasso on the same set of data will possibly generate different set of selected features, which presents challenges for replicating and interpreting the results. To address this problem, several groups proposed to explicitly incorporate the correlation structure into the sparse prediction model and encourage the selection/exclusion of all highly correlated features together [18–21]. Among those is GraphNet, where a graph $G\subseteq R^{p\times p}$ indicating the correlation structure between predictors is used as a priori to guide the feature selection (Eq 2) [21].

$$\begin{array}{c} \min_{w}{∥y-Xw∥}^{2}+\lambda w^{T}{Lw\;\text{s}.\text{t}.\;∥w∥}_{1}\leq t \end{array}$$ (2)

Here, **L** is the corresponding Laplacian matrix of graph **G**. However, GraphNet only accounts for the local topology information with a focus on pairwise similarity. For multi-omic biomarker discovery, using this penalty still can not guarantee the connectivity of selected features in the prior network.

In this paper, we propose a new modularity-constrained Lasso which leverages the global network property to encourage the selection of a sub-network rather than individual markers scattered in the prior network. Given the trans-omic network capturing the functional relationships between SNPs, genes and proteins, we formulate it as a graph and its corresponding adjacency matrix is denoted as $G\subseteq R^{p\times p}$. **B** is the modularity matrix [22], where $B_{ij}=G_{ij}-\frac{h_{i}h_{j}}{2m}$. It evaluates whether the number of links between nodes *i* and *j* is significantly more than expected. *h*_*i*_ and *h*_*j*_ are the degrees of the *i*-th and *j*-th node in the network, and *m* is the total number of links in the network. Inspired by the module identification problem [23, 24], we propose a new penalty term as *P*_*M*_(**w**, **B**) = < **w**^*T*^ **w**, **B**> to impose a modular structure in the identified biomarkers. Here, <> is the Frobenius inner product defined by <*A*, *B* > = *tr*(*A*^*T*^ *B*). Maximizing the Frobenius inner product between **w**^*T*^ **w** and the modularity matrix **B** encourages the selection of features with dense functional connections in the prior multi-omic network. Taken together, our new modularity-constrained Lasso objective is formulated as in Eq 3.

$$\begin{array}{c} \min_{w}\sum_{i=1}^{q}{∥y-Xw∥}^{2}-P_{M}\left(w,B\right){\;\text{s}.\text{t}.\;∥w∥}_{1}\leq t \end{array}$$ (3)

Here, λ and *t* are the parameters that control and balance the contribution from two regularization terms. Note that the objective function in Eq 3 is not convex because the modularity matrix **B** used in *P*_*M*_(**w**, **B**) = < **w**^*T*^ **w**, **B**> is indefinite. To make **B** negative-definite, we introduced an auxiliary function where **B** is replaced by **B** − λ_*B*_ **I** and λ_*B*_ is the absolute maximum eigenvalue of **B**. Eq 3 can be easily solved by obtaining a closed form solution without L1 constraint, followed by soft-thresholding method [17].

## Results

### Performance comparison with competing methods

In this section, we denote our modularity-constrained Lasso as M-Lasso and GraphNet-constrained Lasso as G-Lasso. We compared M-Lasso with three state-of-the-art sparse regression models: G-Lasso, elastic net and traditional Lasso. For M-Lasso and G-Lasso, nested 5-fold cross validation (CV) procedure was applied to tune the parameters. Elastic net and traditional Lasso were both implemented using glmnet Matlab package with 5-fold cross validation. To provide an unbiased estimate of the prediction performance of each method tested in the experiments, all methods were evaluated using the same partition of subjects during the cross validation procedure. The portion of AD, MCI and CN participants was kept the same for each fold. Root Mean Square Error (RMSE) and mean absolute error (MAE) between the predicted values and actual values of all the test subjects were used to compare the prediction performances across different methods.

Prediction performance, measured by RMSE and MAE, of four different regression models on test data set is shown in Table 2. It is observed that, across all 5 folds, M-Lasso largely outperforms G-Lasso, elastic net and traditional Lasso. Traditional Lasso occasionally shows the best performance with smallest prediction error. However, its selected markers are much less connected than those of M-Lasso and G-Lasso due to lack of prior structure constraints and thus will present challenges for further interpretation.

**Table 2:** Performance comparison on test set between M-Lasso and other methods.

		fold 1	fold 2	fold 3	fold 4	fold 5	Mean
RMSE	M-Lasso	**0.852**	1.082	**0.905**	0.959	**0.874**	**0.935**
	G-Lasso	0.876	1.128	1.019	1.178	1.415	1.123
	Elastic Net	0.871	1.073	0.936	0.968	1.577	1.085
	Lasso	0.852	**1.039**	0.931	**0.952**	2.259	1.207
MAE	M-Lasso	**0.699**	0.872	**0.659**	**0.723**	**0.671**	**0.725**
	G-Lasso	0.726	0.893	0.751	0.881	0.872	0.825
	Elastic Net	0.743	0.861	0.687	0.757	0.857	0.781
	Lasso	0.726	**0.831**	0.686	0.758	0.992	0.799

^1^ RMSE: root mean square error;

^2^ MAE: mean absolute error.

For feature selection, M-Lasso identified around 600 features including SNPs, genes and proteins, to be predictive of cognitive performance, while G-Lasso only identified a handful of them (i.e., less than 20 for all 5 folds). When mapped to the prior functional connectivity network, markers identified by G-Lasso scatters across the network with much fewer connections, which suggests that the local topology information used in GraphNet penalty is not strong enough to form subnetwork structure among identified biomarkers. Although the total number of markers identified in G-lasso is much fewer, lack of connections makes them hard to interpret and presents challenges for further functional validation. For M-Lasso, multi-omic biomarkers identified are largely connected to each other in the prior network. Taken together, the sparsity constraints in M-Lasso is still encouraged, but not as strict as in traditional Lasso, elastic net and G-Lasso. Compared to the ridge penalty in elastic net and the GraphNet penalty in G-Lasso, the modularity penalty in M-Lasso further relaxed the sparsity constraint such that subnetworks, instead of individual features, can be identified as biomarkers.

**Fig 2:**
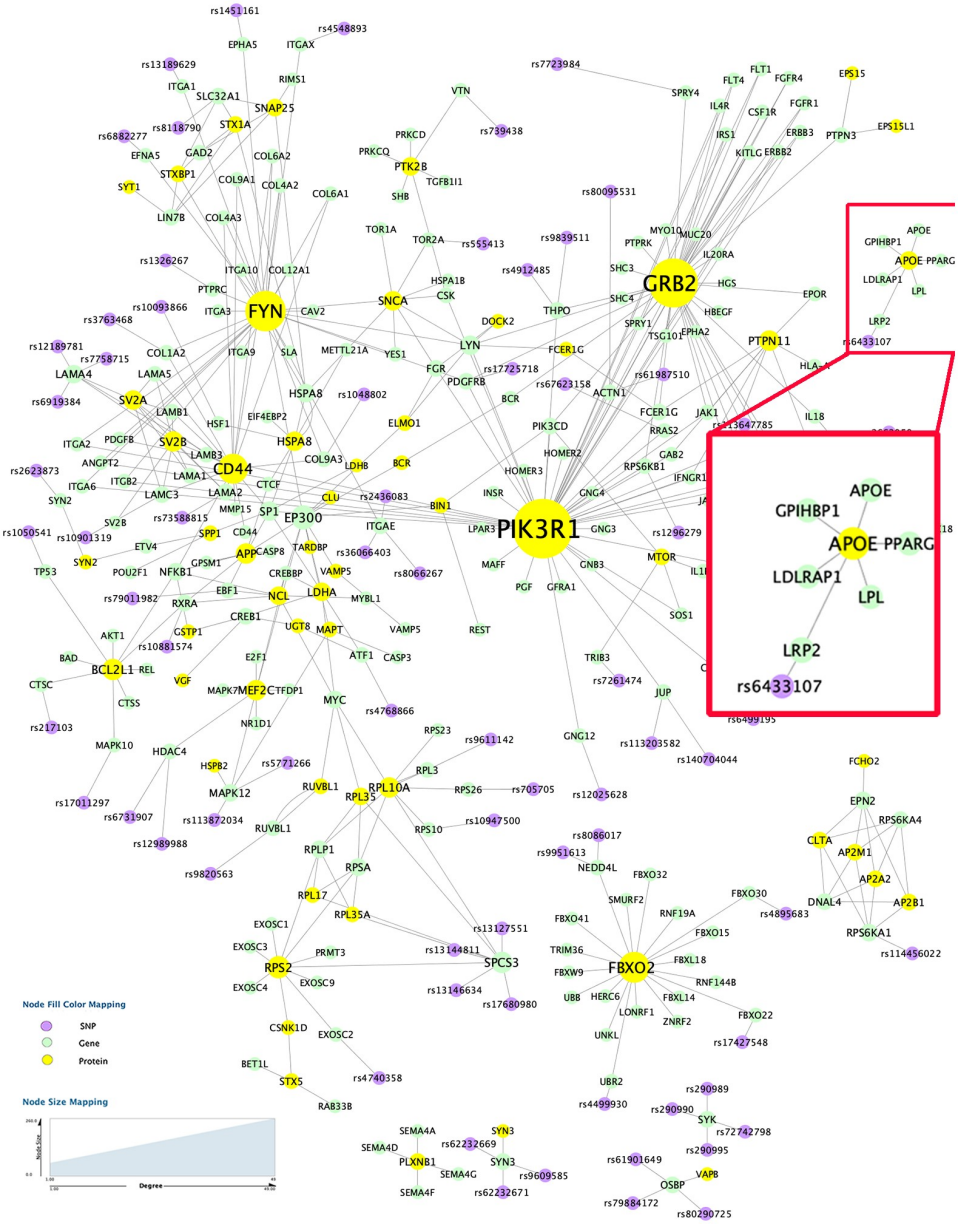
Top 7 connected components with biomarkers identified using M-Lasso mapped to prior network. Inside the red box is the subnetwork involving *APOE* gene, a top risk factor of Alzheimer’s disease.

Take the result from one fold as example, 650 features were selected in M-Lasso, including 255 SNPs, 339 genes and 56 proteins. In particular, there are 7 subnetworks with more than 5 nodes and the largest connected network component involves 276 multi-omic features with 366 edges (Fig 2). The rest of the multi-omic markers identified in M-Lasso mostly form small connected components, ranging in size from 2 to 4. These features are found predictive yet not well functionally connected, possibly due to the fact that they are false positives or their functional connections have not been previously studied yet. In the subsequent part, we focus on the multi-omic biomarkers in the largest connected component, which are not only predictive of cognitive performance but also functionally connected with evidence from prior knowledge.

### Functionally connected multi-omic biomarkers

Shown in Fig 2 are the 7 subnetworks with more than 5 nodes, which were obtained after mapping 650 features back to the prior network. Size of each node is made proportional to their degree. *APOE* gene and its corresponding protein, a top risk factor of AD, is found in one of the subnetworks involving 1 SNP and 5 genes, including *LRP2*, *PPARG*, *LPL*, *LPLRAP1* and *GHIHBP1*. Across all the subnetwoks, we observed that there are multiple trans-omic paths from SNPs to genes and then proteins. Note that these SNPs are located upstream of their connected genes and has significant effect on the transcription factor binding activity. Thus, these SNPs are very likely to have an influence on the expression of their connected genes. Also, the functional interaction between genes and proteins are curated from the REACTOME pathways with direction information. Therefore, genes have a regulatory role toward the expression of their connected proteins in the prior network. Taken together, these trans-omic paths suggest that cognitive performance can be potentially affected by the genetic variations (i.e., SNPs) due to their cascade effect on the expression of downstream genes, which further regulate the protein expression. In addition, we examined 63 SNPs involved in the largest connected component in the BRAINEAC database. This database provides the association between SNPs and gene expression tested on 134 neuropathologically confirmed control individuals of European descent. SNPs significantly associated with gene expression are named as expression quantitative trait loci (eQTLs). EQTL mapping is a widely used tool for identifying genetic variants that affect gene regulation [25]. Details of the eQTL analysis can be found in [26]. For 63 SNPs in the largest subnetwork, 58 of them were found to be eQTLs in the frontal cortex tissue (FDR corrected *p* < 0.05).

For the largest connected subnetwork, we further performed network analysis using NetworkAnalyzer in Cytoscape [27] and identified the biomarkers with top centrality values, such as degree, betweenness and closeness (Table 3). Top nodes by degree in this subnetwork included proteins *PIK3R1*, *GRB2*, *FYN*, *CD44*, *RPS2*, *BCL2L1*, *BCL2L1* and *PTPN11*, and genes *EP300* and *SPCS3*. Most hub nodes are also found to have the top centrality value in betweenness and closeness, such as *PIK3R1*, *FYN*, and *EP300*. Majority of these genes and proteins have been previously reported in association with AD. For example, *PIK3R1* encodes the regulatory subunit of the phosphoinositide-3-kinase protein complex PI3Ks, which are known to play a key role in insulin signaling. Results from recent studies start to show evidence of intrinsic insulin resistance inside AD brains [28]. The hub gene *EP300* encodes the enzyme histone acetyltransferase *P300* or E1A-associated protein *P300*. This enzyme functions as histone acetyltransferase that regulates transcription of genes via chromatin remodeling. Findings from multiple studies have suggested the potential of *P300* to act as a biomarker for dementia assessment and monitoring AD [29, 30]. In addition, *GRB2* was found to interact with *APP*, a well-known gene related to AD. *GRB2* interacts with *APP* requiring phosphorylation of *APP* at Tyr-682 [31]. This could lead to the activation of the MAPK pathway, since *GRB2* are known to link growth factor receptors to signaling pathways, such as *MAPK* and *PI3K*, and participate in oncogenic proliferation, neuronal development, cell differentiation, and apoptosis [32–37]. In addition to rank, a subsampling procedure can help determine a hard cut-off threshold for identification of a smaller set of significant features [38]. For example, we can repeatedly select the same number of random features, map them to the prior network and use the average (or 25% percentile) of the derived network centrality values as threshold to select significant features.

**Table 3:** Nodes with top centrality values in the largest connected subnetwork.

Degree	*PIK3R1*, *GRB2*, *FYN*, *CD44*, ***EP300***, *RPS2*, ***SPCS3*** *BCL2L1*, *RPL10A*, *PTPN11*
Average Shortest Path Length	*PIK3R1*, ***SP1***, *FYN*, ***LYN***, *LDHA*, ***PDGFRB***, ***EP300***, *APP*, ***CSK***, *CD44*
Betweenness	*PIK3R1*, ***SP1***, *FYN*, ***EP300***, ***MYC***, *GRB2*, *LDHA*, *CD44*, *RPL10A*, ***LYN***
Closeness	*PIK3R1*, ***SP1***, *FYN*, ***LYN***, *LDHA*, ***PDGFRB***, ***EP300***, *APP*, ***CSK***, *CD44*

^1^ rs- IDs: SNPs; Bold: genes; The rest: proteins.

Finally, hub genes are known to be likely disease-associated genes. Features with higher degree in the identified subnetworks are expected to be more important with higher absolute regression weights. Therefore, we further tested the association between degree of features and their absolute regression weights derived from M-Lasso. Across 5 folds, the average Pearson’s correlation value between node degree and absolute weight is only 0.18. However, when we excluded the features with low degree (degree <3), the average correlation increases significantly to 0.4. This indicates that the importance of identified multi-omic features is more proportional to their degrees only when they already have many interactions in the prior network. Upon further examination, features with high degree were found to have medium to high regression weights. Features with low degree can have very small or very high weights. This is possibly due to the fact that their low degree may be a result of no interaction or no known interaction. Therefore, their importance is less determined than that of hub ones.

### Pathway enrichment analysis

For 166 genes, 47 proteins and 63 SNPs in the largest connected subnetwork, we performed pathway enrichment analysis based on the Kyoto Encyclopedia of Genes and Genomes (KEGG) database [39]. The enrichment analysis was performed using ClueGO as left-sided tests based on the hypergeometric distribution [40]. In total, 77 pathways were found to be significantly enriched by our gene/protein set, with Bonferroni corrected term p-value smaller than 5% (corrected *p* ≤ 0.05). Shown in Table 4 was the top 20 enriched KEGG pathways with smallest p values after correction. The top hit is PI3K-Akt signaling pathway, a major mediator of effects of insulin. Two recent studies have found a significant correlation between peripheral insulin resistance and brain A*β* levels as measured by Pittsburgh compound B-positron emission tomography (PiB-PET) [41, 42]. The impaired insulin-PI3K-Akt signaling observed in the AD brain has led to clinical trials studying whether the enhancement of this pathway using intranasal insulin (IN) treatment is beneficial [43]. Other enriched pathways that are previously reported with a key role in AD include Focal adhesion [44], ECM-receptor interaction [45], Ras signaling pathway [46], *MAPK* signaling pathway [47], Rap1 signaling pathway [48], etc. Many of the top enriched pathways are related to cancer, such as PI3K-Akt signaling pathway, prostate cancer and small lung cancer. In addition, we performed pathway enrichment analysis for the subnetwork involving *APOE* gene and protein. Interestingly, the cholesterol metabolism pathway was significantly enriched by these genes and proteins. This provides support to recent findings in the association between cholesterol metabolism and memory performance [49–51].

**Table 4:** Top enriched KEGG pathways by the genes and proteins in the largest connected subnetwork.

Pathway	# of markers in the pathway	# of genes in the pathway	p-value	Corrected p-value
PI3K-Akt signaling pathway	64	354	2.24E-36	3.39E-34
Focal adhesion	41	199	2.81E-25	4.22E-23
Pathways in cancer	59	530	8.78E-22	1.31E-19
ECM-receptor interaction	25	82	4.04E-20	5.97E-18
Human papillomavirus infection	42	330	1.54E-17	2.26E-15
Ras signaling pathway	34	232	3.65E-16	5.34E-14
Small cell lung cancer	22	93	3.12E-15	4.53E-13
MAPK signaling pathway	36	295	1.65E-14	2.38E-12
Kaposi sarcoma-associated herpesvirus infection	27	186	6.77E-13	9.67E-11
Toxoplasmosis	21	113	2.20E-12	3.13E-10
Prostate cancer	18	97	9.14E-11	1.29E-08
Chronic myeloid leukemia	16	76	1.45E-10	2.03E-08
Phospholipase D signaling pathway	21	148	4.49E-10	6.25E-08
Amoebiasis	17	96	6.70E-10	9.25E-08
Human cytomegalovirus infection	25	225	1.83E-09	2.51E-07
Relaxin signaling pathway	19	130	1.91E-09	2.60E-07
Acute myeloid leukemia	14	66	1.93E-09	2.60E-07
Proteoglycans in cancer	23	201	4.84E-09	6.49E-07
ErbB signaling pathway	15	85	7.42E-09	9.87E-07
Rap1 signaling pathway	23	206	7.81E-09	1.03E-06

## Conclusion

In this study, we proposed a new modularity-constrained Lasso model to jointly analyze the genotype, RNA-Seq gene expression and protein expression data. The newly introduced penalty term maximizes the global modularity of selected biomarkers in the prior network and encourages the selection of multi-omic biomarkers forming network modules. With this new penalty term, M-Lasso is advantageous in that features can be selected either because they are predictive or because they are closely connected with many predictive ones in the prior network. Thus, the sparsity constraint in M-Lasso is much more relaxed than in G-Lasso, elastic net and traditional Lasso. Compared to the GraphNet penalty that enforces local pairwise similarity, modularity-based penalty helps identify more biomarkers with significantly improved functional connectivity. In particular, we found that some biomarkers form trans-omic paths from SNP to gene and then protein, suggesting the potential cascade effect of genetic variations on the downstream transcriptome and proteome level. To the best of our knowledge, this is the first study that explored the potential of functional multi-omic subnetworks as biomarkers in AD.

Despite the promising findings, the proposed M-Lasso still has multiple limitations. First, only one disease quantitative trait is used as outcome in the prediction model. Considering the potential bias introduced in data collection procedure, the biomarkers and their functional connectivity network identified here may not reflect the optimal pattern. Incorporating multiple correlated outcomes and performing a multitask prediction will possibly help improve the performance. Second, like many multi-view prediction models, the proposed M-Lasso is not very capable in handling the missing data problem. Each subject has to have all types of data to be included in the analysis. Therefore, subjects with missing data in one or more data types are inevitably excluded. This missing data problem can be partly addressed using imputation methods such as singular value decomposition [52] and matrix completion [53]. In case of subjects with large chunk of missing data, one possible solution is to examine two types of data at a time to maximize the number of available subjects. Future efforts are in need to further improve this model to enable the integrative analysis of multi-view data from decoupled subjects.

## Supporting information

S1 File (XLSX)

## References

[1] Organization WH, et al Global Health Estimates 2016: Deaths by Cause. Age, Sex, by Country and by Region. 2000;2016:2018.

[2] Visscher PM, Brown MA, McCarthy MI, Yang J. Five years of GWAS discovery. The American Journal of Human Genetics. 2012;90(1):7–24. 10.1016/j.ajhg.2011.11.029 22243964 PMC3257326

[3] Edwards SL, Beesley J, French JD, Dunning AM. Beyond GWASs: illuminating the dark road from association to function. The American Journal of Human Genetics. 2013;93(5):779–797. 10.1016/j.ajhg.2013.10.012 24210251 PMC3824120

[4] Mueller SG, Weiner MW, Thal LJ, Petersen RC, Jack C, Jagust W, et al The Alzheimer’s disease neuroimaging initiative. Neuroimaging Clinics. 2005;15(4):869–877. 10.1016/j.nic.2005.09.008 16443497 PMC2376747

[5] A Bennett D, A Schneider J, Arvanitakis Z, S Wilson R. Overview and findings from the religious orders study. Current Alzheimer Research. 2012;9(6):628–645. 10.2174/156720512801322573 22471860 PMC3409291

[6] Hasin Y, Seldin M, Lusis A. Multi-omics approaches to disease. Genome biology. 2017;18(1):83 10.1186/s13059-017-1215-1 28476144 PMC5418815

[7] Huang S, Chaudhary K, Garmire LX. More is better: recent progress in multi-omics data integration methods. Frontiers in genetics. 2017;8:84 10.3389/fgene.2017.00084 28670325 PMC5472696

[8] De Jager PL, Shulman JM, Chibnik LB, Keenan BT, Raj T, Wilson RS, et al A genome-wide scan for common variants affecting the rate of age-related cognitive decline. Neurobiology of aging. 2012;33(5):1017–e1. 10.1016/j.neurobiolaging.2011.09.033 22054870 PMC3307898

[9] Horgusluoglu-Moloch E, Nho K, Risacher SL, Kim S, Foroud T, Shaw LM, et al Targeted neurogenesis pathway-based gene analysis identifies ADORA2A associated with hippocampal volume in mild cognitive impairment and Alzheimer’s disease. Neurobiology of aging. 2017;60:92–103. 10.1016/j.neurobiolaging.2017.08.010 28941407 PMC5774672

[10] Nho K, Corneveaux J, Kim S, Lin H, Risacher S, Shen L, et al Whole-exome sequencing and imaging genetics identify functional variants for rate of change in hippocampal volume in mild cognitive impairment. Molecular psychiatry. 2013;18(7):781 10.1038/mp.2013.24 23608917 PMC3777294

[11] Petyuk VA, Qian WJ, Smith RD, Smith DJ. Mapping protein abundance patterns in the brain using voxelation combined with liquid chromatography and mass spectrometry. Methods. 2010;50(2):77–84. 10.1016/j.ymeth.2009.07.009 19654045 PMC2818068

[12] Andreev VP, Petyuk VA, Brewer HM, Karpievitch YV, Xie F, Clarke J, et al Label-free quantitative LC–MS proteomics of Alzheimer’s disease and normally aged human brains. Journal of proteome research. 2012;11(6):3053–3067. 10.1021/pr3001546 22559202 PMC3445701

[13] Fabregat A, Jupe S, Matthews L, Sidiropoulos K, Gillespie M, Garapati P, et al The reactome pathway knowledgebase. Nucleic acids research. 2017;46(D1):D649–D655. 10.1093/nar/gkx1132 PMC5753187 29145629

[14] Kumar S, Ambrosini G, Bucher P. SNP2TFBS–a database of regulatory SNPs affecting predicted transcription factor binding site affinity. Nucleic acids research. 2016;45(D1):D139–D144. 10.1093/nar/gkw1064 27899579 PMC5210548

[15] Folstein MF, Folstein SE, McHugh PR. “Mini-mental state”: a practical method for grading the cognitive state of patients for the clinician. Journal of psychiatric research. 1975;12(3):189–198. 10.1016/0022-3956(75)90026-6 1202204

[16] Folstein MF, Robins LN, Helzer JE. The mini-mental state examination. Archives of general psychiatry. 1983;40(7):812–812. 10.1001/archpsyc.1983.01790060110016 6860082

[17] Tibshirani R. Regression shrinkage and selection via the lasso. Journal of the Royal Statistical Society: Series B (Methodological). 1996;58(1):267–288.

[18] Jacob L, Obozinski G, Vert JP. Group lasso with overlap and graph lasso. In: Proceedings of the 26th annual international conference on machine learning. ACM; 2009. p. 433–440.

[19] Yuan L, Liu J, Ye J. Efficient methods for overlapping group lasso. In: Advances in Neural Information Processing Systems; 2011 p. 352–360.

[20] Kim S, Xing EP, et al Tree-guided group lasso for multi-response regression with structured sparsity, with an application to eQTL mapping. The Annals of Applied Statistics. 2012;6(3):1095–1117. 10.1214/12-AOAS549

[21] Grosenick L, Klingenberg B, Katovich K, Knutson B, Taylor JE. Interpretable whole-brain prediction analysis with GraphNet. NeuroImage. 2013;72:304–321. 10.1016/j.neuroimage.2012.12.062 23298747

[22] Newman ME. Modularity and community structure in networks. Proceedings of the national academy of sciences. 2006;103(23):8577–8582. 10.1073/pnas.0601602103 PMC1482622 16723398

[23] Hildebrand R. Identification of community structure in networks with convex optimization. arXiv preprint arXiv:08061896. 2008;.

[24] Chan YK, Yeung DY. A convex formulation of modularity maximization for community detection. In: Twenty-Second International Joint Conference on Artificial Intelligence; 2011.

[25] Gilad Y, Rifkin SA, Pritchard JK. Revealing the architecture of gene regulation: the promise of eQTL studies. Trends in genetics. 2008;24(8):408–415. 10.1016/j.tig.2008.06.001 18597885 PMC2583071

[26] Ramasamy A, Trabzuni D, Guelfi S, Varghese V, Smith C, Walker R, et al Genetic variability in the regulation of gene expression in ten regions of the human brain. Nature neuroscience. 2014;17(10):1418–1428. 10.1038/nn.3801 25174004 PMC4208299

[27] Shannon P, Markiel A, Ozier O, Baliga NS, Wang JT, Ramage D, et al Cytoscape: a software environment for integrated models of biomolecular interaction networks. Genome research. 2003;13(11):2498–2504. 10.1101/gr.1239303 14597658 PMC403769

[28] Arnold SE, Arvanitakis Z, Macauley-Rambach SL, Koenig AM, Wang HY, Ahima RS, et al Brain insulin resistance in type 2 diabetes and Alzheimer disease: concepts and conundrums. Nature Reviews Neurology. 2018;14(3):168 10.1038/nrneurol.2017.185 29377010 PMC6098968

[29] Polich J, Ladish C, Bloom FE. P300 assessment of early Alzheimer’s disease. Electroencephalography and Clinical Neurophysiology/Evoked Potentials Section. 1990;77(3):179–189. 10.1016/0168-5597(90)90036-D 1691970

[30] Hedges D, Janis R, Mickelson S, Keith C, Bennett D, Brown BL. P300 amplitude in Alzheimer’s disease: a meta-analysis and meta-regression. Clinical EEG and neuroscience. 2016;47(1):48–55. 10.1177/1550059414550567 25253434

[31] Roncarati R, Šestan N, Scheinfeld MH, Berechid BE, Lopez PA, Meucci O, et al The *γ*-secretase-generated intracellular domain of *β*-amyloid precursor protein binds Numb and inhibits Notch signaling. Proceedings of the National Academy of Sciences. 2002;99(10):7102–7107. 10.1073/pnas.102192599 12011466 PMC124535

[32] Russo C, Dolcini V, Salis S, Venezia V, Zambrano N, Russo T, et al Signal transduction through tyrosine-phosphorylated C-terminal fragments of amyloid precursor protein via an enhanced interaction with Shc/Grb2 adaptor proteins in reactive astrocytes of Alzheimer’s disease brain. Journal of Biological Chemistry. 2002;277(38):35282–35288. 10.1074/jbc.M110785200 12084708

[33] Cattaneo E, Pelicci PG. Emerging roles for SH2/PTB-containing Shc adaptor proteins in the developing mammalian brain. Trends in neurosciences. 1998;21(11):476–481. 10.1016/S0166-2236(98)01282-X 9829689

[34] Yokote K, Mori S, Hansen K, McGlade J, Pawson T, Heldin CH, et al Direct interaction between Shc and the platelet-derived growth factor beta-receptor. Journal of Biological Chemistry. 1994;269(21):15337–15343. 8195171

[35] Saucier C, Papavasiliou V, Palazzo A, Naujokas MA, Kremer R, Park M. Use of signal specific receptor tyrosine kinase oncoproteins reveals that pathways downstream from Grb2 or Shc are sufficient for cell transformation and metastasis. Oncogene. 2002;21(12):1800 10.1038/sj.onc.1205261 11896612

[36] Napoli C, Martin-Padura I, De Nigris F, Giorgio M, Mansueto G, Somma P, et al Deletion of the p66Shc longevity gene reduces systemic and tissue oxidative stress, vascular cell apoptosis, and early atherogenesis in mice fed a high-fat diet. Proceedings of the National Academy of Sciences. 2003;100(4):2112–2116. 10.1073/pnas.0336359100 PMC149967 12571362

[37] Dankort D, Maslikowski B, Warner N, Kanno N, Kim H, Wang Z, et al Grb2 and Shc adapter proteins play distinct roles in Neu (ErbB-2)-induced mammary tumorigenesis: implications for human breast cancer. Molecular and cellular biology. 2001;21(5):1540–1551. 10.1128/MCB.21.5.1540-1551.2001 11238891 PMC86700

[38] Wu CC, Kannan K, Lin S, Yen L, Milosavljevic A. Identification of cancer fusion drivers using network fusion centrality. Bioinformatics. 2013;29(9):1174–1181. 10.1093/bioinformatics/btt131 23505294 PMC3634186

[39] Kanehisa M, Goto S. KEGG: kyoto encyclopedia of genes and genomes. Nucleic acids research. 2000;28(1):27–30. 10.1093/nar/28.1.27 10592173 PMC102409

[40] Bindea G, Mlecnik B, Hackl H, Charoentong P, Tosolini M, Kirilovsky A, et al ClueGO: a Cytoscape plug-in to decipher functionally grouped gene ontology and pathway annotation networks. Bioinformatics. 2009;25(8):1091–1093. 10.1093/bioinformatics/btp101 19237447 PMC2666812

[41] Willette AA, Johnson SC, Birdsill AC, Sager MA, Christian B, Baker LD, et al Insulin resistance predicts brain amyloid deposition in late middle-aged adults. Alzheimer’s & dementia. 2015;11(5):504–510. 10.1016/j.jalz.2014.03.011 PMC4297592 25043908

[42] Ekblad LL, Johansson J, Helin S, Viitanen M, Laine H, Puukka P, et al Midlife insulin resistance, APOE genotype, and late-life brain amyloid accumulation. Neurology. 2018;90(13):e1150–e1157. 10.1212/WNL.0000000000005214 29476033 PMC5880630

[43] Gabbouj S, Ryhänen S, Marttinen M, Wittrahm R, Takalo M, Kemppainen S, et al Altered insulin signaling in Alzheimer’s disease brain–special emphasis on PI3K-Akt pathway. Frontiers in neuroscience. 2019;13:629 10.3389/fnins.2019.00629 31275108 PMC6591470

[44] Caltagarone J, Jing Z, Bowser R. Focal adhesions regulate A*β* signaling and cell death in Alzheimer’s disease. Biochimica et Biophysica Acta (BBA)-Molecular Basis of Disease. 2007;1772(4):438–445. 10.1016/j.bbadis.2006.11.007 17215111 PMC1876750

[45] Kerrisk ME, Cingolani LA, Koleske AJ. ECM receptors in neuronal structure, synaptic plasticity, and behavior In: Progress in brain research. vol. 214 Elsevier; 2014 p. 101–131. 25410355 10.1016/B978-0-444-63486-3.00005-0 PMC4640673

[46] Kirouac L, Rajic AJ, Cribbs DH, Padmanabhan J. Activation of Ras-ERK signaling and GSK-3 by amyloid precursor protein and amyloid beta facilitates neurodegeneration in Alzheimer’s disease. Eneuro. 2017;4(2). 10.1523/ENEURO.0149-16.2017 PMC5367084 28374012

[47] Zhu X, Lee Hg, Raina AK, Perry G, Smith MA. The role of mitogen-activated protein kinase pathways in Alzheimer’s disease. Neurosignals. 2002;11(5):270–281. 10.1159/000067426 12566928

[48] Dumbacher M, Van Dooren T, Princen K, De Witte K, Farinelli M, Lievens S, et al Modifying Rap1-signalling by targeting Pde6*δ* is neuroprotective in models of Alzheimer’s disease. Molecular neurodegeneration. 2018;13(1):50 10.1186/s13024-018-0283-3 30257685 PMC6158915

[49] Engelman CD, Koscik RL, Jonaitis EM, Okonkwo OC, Hermann BP, La Rue A, et al Interaction between two cholesterol metabolism genes influences memory: findings from the Wisconsin Registry for Alzheimer’s Prevention. Journal of Alzheimer’s disease. 2013;36(4):749–757. 10.3233/JAD-130482 23669301 PMC3759032

[50] Ali-Rahmani F, Grigson PS, Lee S, Neely E, Connor JR, Schengrund CL. H63D mutation in hemochromatosis alters cholesterol metabolism and induces memory impairment. Neurobiology of aging. 2014;35(6):1511–e1. 10.1016/j.neurobiolaging.2013.12.014 24439478

[51] Pérez-Cañamás A, Sarroca S, Melero-Jerez C, Porquet D, Sansa J, Knafo S, et al A diet enriched with plant sterols prevents the memory impairment induced by cholesterol loss in senescence-accelerated mice. Neurobiology of aging. 2016;48:1–12. 10.1016/j.neurobiolaging.2016.08.009 27622776

[52] Schneider T. Analysis of incomplete climate data: Estimation of mean values and covariance matrices and imputation of missing values. Journal of climate. 2001;14(5):853–871. 10.1175/1520-0442(2001)014<0853:AOICDE>2.0.CO;2

[53] Thung KH, Wee CY, Yap PT, Shen D, Initiative ADN, et al Neurodegenerative disease diagnosis using incomplete multi-modality data via matrix shrinkage and completion. NeuroImage. 2014;91:386–400. 10.1016/j.neuroimage.2014.01.033 24480301 PMC4096013

